# Performance Analysis of Relative GPS Positioning for Low-Cost Receiver-Equipped Agricultural Rovers

**DOI:** 10.3390/s23218835

**Published:** 2023-10-30

**Authors:** Gustavo S. Carvalho, Felipe O. Silva, Marcus Vinicius O. Pacheco, Gleydson A. O. Campos

**Affiliations:** 1 Department of Automatics, Federal University of Lavras, Lavras 37203-202, Brazil; guhcarv93@gmail.com (G.S.C.); marcuspachecomvp@gmail.com (M.V.O.P.); 2 Department of Agricultural Engineering, Federal University of Lavras, Lavras 37203-202, Brazil; gleydson.campos@ufla.br

**Keywords:** RGNSS, communication failure, baseline separation, positioning accuracy, precision agriculture, moving rover

## Abstract

Global navigation satellite systems (GNSSs) became an integral part of all aspects of our lives, whether for positioning, navigation, or timing services. These systems are central to a range of applications including road, aviation, maritime, and location-based services, agriculture, and surveying. The Global Positioning System (GPS) Standard Position Service (SPS) provides position accuracy up to 10 m. However, some modern-day applications, such as precision agriculture (PA), smart farms, and Agriculture 4.0, have demanded navigation technologies able to provide more accurate positioning at a low cost, especially for vehicle guidance and variable rate technology purposes. The Society of Automotive Engineers (SAE), for instance, through its standard J2945 defines a maximum of 1.5 m of horizontal positioning error at 68% probability (1σ), aiming at terrestrial vehicle-to-vehicle (V2V) applications. GPS position accuracy may be improved by addressing the common-mode errors contained in its observables, and relative GNSS (RGNSS) is a well-known technique for overcoming this issue. This paper builds upon previous research conducted by the authors and investigates the sensitivity of the position estimation accuracy of low-cost receiver-equipped agricultural rovers as a function of two degradation factors that RGNSS is susceptible to: communication failures and baseline distances between GPS receivers. The extended Kalman filter (EKF) approach is used for position estimation, based on which we show that it is possible to achieve 1.5 m horizontal accuracy at 68% probability (1σ) for communication failures up to 3000 s and baseline separation of around 1500 km. Experimental data from the Brazilian Network for Continuous Monitoring of GNSS (RBMC) and a moving agricultural rover equipped with a low-cost GPS receiver are used to validate the analysis.

## 1. Introduction

Over the last several decades, global navigation satellite systems (GNSSs) [1,2] have become the dominant approach for personal and vehicular (terrestrial or aerial) navigation [3,4,5,6,7,8]. GNSSs are “position-fixing” systems that allow a position and timing solution to be computed from synchronized ranging signals broadcast by satellites [9,10]. They provide three types of measurements, known as observables [9,11,12,13]: pseudoranges (coarse distances between satellites and receivers during the transmission and reception of GNSS signals), Doppler shifts (associated with the relative speed of the satellite-receiver pairs), and carrier phases (fine distances between the satellites and receivers expressed in the unit of cycles of the carrier wave). The GNSS navigation solution can meet many application specifications using low-cost receivers, and the resulting position errors are bounded. However, standalone GNSS (SGNSS) does not meet the necessary performance requirements of position accuracy and reliability in the new generation of applications (e.g., connected autonomous vehicles (CAVs), vehicle-to-vechicle (V2V) communication, driver’s assistance, precision agriculture (PA), smart farms, and Agriculture 4.0) [14,15,16,17], since their signals have low power and, consequently, are vulnerable to numerous types of errors.

In the field of PA, the acquisition of precise position data for the effective control and analysis of a huge amount of geospatial information is of paramount importance [18,19]. This is an approach to farm management that uses information technology to deal with the spatial variability present in farmland, ensuring that crops and soil receive exactly what they need for optimum health and productivity [20]. GNSS-based applications in precision farming are largely being used for farm planning, field mapping, soil sampling, tractor guidance, crop scouting, variable rate applications, and yield mapping [21,22,23,24,25,26].

Currently, GNSSs comprise four constellations [9,12]: the Global Positioning System (GPS), the oldest and most popular system, which has been operating with full operational capability (FOC) since 1995 under the government of the United States of America [27]; GLONASS, the Russian system with FOC dated from late 1990s [28]; BeiDou, the Chinese constellation which reached FOC in 2020 [29]; and Galileo, the European constellation and a unique system that is under civilian control [30].

Pseudorange measurements (the main GNSS observables) are primarily corrupted by two categories of errors [27,31,32]:

- Common-Mode Errors (CME): These are spatially and temporally correlated errors (i.e., they comprise propagation and temporal systematic errors that are experienced by all receivers in the same vicinity and time span). Among them, ephemeris error, satellite clock bias, intersignal biases, and ionospheric and tropospheric delays stand out.
- Non-Common-Mode Errors (NCME): Unlike CMEs, NCMEs are different for each receiver, mainly comprising receiver clock bias, multipath error, and receiver tracking noise.

The GPS Standard Position Service (SPS) provides horizontal and vertical accuracy of 9 m and 15 m, respectively [33]. However, specifications such as the J2945 standard [34], proposed by the Society of Automotive Engineers (SAE), which is aimed at vehicle-to-vehicle (V2V) applications, require horizontal and vertical accuracy of 1.5 m and 3 m, respectively, with 68% probability. Common-mode errors are the main errors responsible for deterioration of the SGNSS position accuracy, and therefore advanced GNSS techniques are required for achieving such a level of accuracy. A number of solutions focused on mitigating the effect of common-mode errors on GNSS position estimation are available in the literature [35,36,37,38,39]. The differential GNSS (DGNSS) approach is a well-known technique which has been largely employed and researched by the civilian community since the beginning of the GNSS era (while selective availability was still activated) [40,41,42,43,44]. Put simply, the technique involves accurately measuring the errors in the pseudoranges observed by a receiver at a known time and location, namely a reference station, and broadcasting these corrections to a rover receiver at an unknown and possibly changing position in the reference base’s vicinity. These “differential corrections” are then applied to the pseudoranges measured by the second receiver so that any errors which are common to the two receivers are eliminated.

As another option for GNSS common-mode error mitigation, relative GNSS (RGNSS) positioning has also been employed in the last few years, boosted by cutting-edge communication technologies supporting high-speed and low-latency data dissemination between GNSS receiver networks [45,46,47,48,49]. The RGNSS technique is based on the sharing of GNSS pseudorange measurements observed in a reference station (i.e., raw observables) with a rover in the vicinity in such a way that these raw data are differentiated from the moving GNSS receiver’s own measurements. In sequence, the rover uses such differentiated observations thorugh position estimation algorithms, aiming at computing its position relative to the reference station. As the reference station location is generally known at the millimeter level, accurate computation of the rover’s absolute position is straightforward.

In GNSS systems that rely on reference stations, such as RGNSS and DGNSS, two primary factors contribute to position accuracy impairment [50]. These factors are failures in communication, which obligates the rover to use outdated corrections (in the case of DGNSS) or raw observables (in the case of RGNSS), and the baseline separation (i.e., the physical distance between receivers). Communication failures in RGNSS position estimation accuracy were investigated in [49] for a static reference station equipped with a high-performance GNSS receiver, demonstrating that RGNSS positioning performance is insensitive (i.e., the position accuracy remains submetric) to failures up to 1500 s. In [48] the effects of baseline separation on RGNSS position accuracy (again for a static reference station equipped with a high-performance GNSS receiver) were in turn investigated, showing that horizontal position errors remain submetric (1σ) for baseline distances up to 2100 km. Similar investigations, devoted to moving (especially agricultural) rovers equipped with low-cost GNSS apparatuses, however, are still rare in the literature [51].

Given these open issues, this paper aims at analyzing the aforementioned degrading factors (i.e., the effect of communication failures and baseline separation on relative GPS (RGPS)) position estimation accuracy for moving agricultural rovers equipped with a low-cost GNSS receiver. The main contribution of this work is a comprehensive analysis concerning the maximum communication failure time and maximum distance between the rover and reference station that allows RGNSS horizontal position accuracy based on a low-cost receiver to achieve the J2945 standard specifications. This work focuses on the results from the GPS constellation only. Hence, the terms GPS and GNSS are used interchangeably hereinafter.

The remainder of this paper is organized as follows. Section 2 reviews the SGNSS and RGNSS approaches, defines the appropriate notation, and presents the corresponding algorithms for position estimation using pseudorange measurements. Section 3 describes the experimental data acquisition set-up, while Section 4 presents the results for the communication failure and baseline separation effects on position accuracy. Lastly, Section 5 summarizes the paper and presents final thoughts and conclusions.

## 2. GNSS Position Estimation

The pseudorange measurement between a rover *a* and a satellite *s*, taking into account the errors cited in Section 1, can be modeled as (time index *t* is omitted for the sake of simplicity):

(1) $$\begin{array}{c} \rho_{a}^{s}=r_{as}+\delta \rho_{c}^{a}-\delta \rho_{c}^{s}+\delta \rho_{I,a}^{s}+\delta \rho_{T,a}^{s}+\delta \rho_{E}^{s}+\delta \rho_{M,a}^{s}+w_{p,a}^{s}, \end{array}$$

where the term $\delta \rho_{c}^{a}$ represents the receiver clock bias, $\delta \rho_{c}^{s}$ is the satellite clock bias, $\delta \rho_{I,a}^{s}$ is the ionospheric delay, $\delta \rho_{T,a}^{s}$ is the tropospheric delay, $\delta \rho_{E}^{s}$ is the ephemeris error, $\delta \rho_{M,a}^{s}$ is the multipath error, $w_{\rho ,a}^{s}$ is a white Gaussian-like measurement noise representing the lumped effects of tracking noise and unmodeled residual errors, and $r_{as}$ is the true range (Euclidean distance) from the Earth-centered, Earth-fixed (ECEF) position of a satellite *s* ($r_{es}^{e}$) to the ECEF position of a rover *a* ($r_{ea}^{e}$), which is defined as

(2) $$\begin{array}{cc} r_{as} & =∥C_{e}^{I}r_{es}^{e}-r_{ea}^{e}∥, \end{array}$$

where $C_{e}^{I}$ is the coordinate transformation matrix (CTM) that compensates for the Sagnac effect during GNSS signal propagation [9]. In this paper, the ECEF frame is represented by the superscript *e* and is defined with its $z_{e}$ axis pointing along the Earth’s axis of rotation, with the $x_{e}$ axis pointing from the center of Earth to the intersection of the equator with the Greenwich meridian and the $y_{e}$ axis completing the right-hand orthogonal set.

The least squares (LS) estimator is a well-known approach commonly used for receiver position computation based on pseudoranges [52,53,54]. On the other hand, a more robust approach for estimating position solutions is provided by a Kalman filter-based estimation algorithm, which incorporates information obtained from previous measurements. Among these filters, the extended Kalman filter (EKF) is widely employed in such applications due to its reliability and relatively low computational requirements [9,12,13,48,55,56]. When utilizing an EKF algorithm for SGNSS positioning estimation, the following state vector is commonly defined:

(3) $$\begin{array}{cc} x_{S}^{e} & ={\left[{\left(r_{ea}^{e}\right)}^{T},{\left(v_{ea}^{e}\right)}^{T},\delta \rho_{c}^{a},\delta {\dot{\rho }}_{c}^{a}\right]}^{T}\in R^{n}, \end{array}$$

where the subscript S denotes SGNSS, n=8, $v_{ea}^{e}$ is the rover velocity, and $\delta {\dot{\rho }}_{c}^{a}$ is the receiver clock drift.

The propagation of the states in Equation (3) over time is governed by the following dynamic model:

(4) $$\begin{array}{cc} {\dot{r}}_{ea}^{e} & =v_{ea}^{e},\;\;\;\;\frac{\partial }{\partial t}\delta \rho_{c}^{a}=\delta {\dot{\rho }}_{c}^{a}\\ {\dot{v}}_{ea}^{e} & =0,\;\;\;\;\;\;\;\frac{\partial }{\partial t}\delta {\dot{\rho }}_{c}^{a}=0 \end{array}$$

The primary factors contributing to increased uncertainty in the state estimates are the user motion, random walk in the receiver clock drift, and phase noise affecting the clock bias. The acceleration power spectral density (PSD) matrix that drives the rover velocity random walk is

(5) $$\begin{array}{c} S_{a}^{e}={\left(C_{e}^{n}\right)}^{T}\left(\begin{array}{ccc} S_{aH} & 0 & 0\\ 0 & S_{aH} & 0\\ 0 & 0 & S_{aV} \end{array}\right)C_{e}^{n}, \end{array}$$

where $C_{e}^{n}$ is the ECEF to the north, east, and down (NED) CTM and $S_{aH}$ and $S_{aV}$ are the horizontal and vertical acceleration PSDs, respectively. In this paper, the NED frame is represented by the superscript *n* and is defined with its origin at the center of mass of the rover, with its $x_{n}$, $y_{n}$, and $z_{n}$ axes pointing in the north, east, and down directions, respectively. The system noise is inherently context-dependent. For a scenario encompassing a moving agricultural rover (low-dynamic application), for instance, $S_{aH}$ and $S_{aV}$ can be tuned as 1 m${}^{2}$s${}^{-3}$. The receiver clock frequency drift PSD and clock phase drift PSD, in turn, can be tuned as $S_{cf}^{a}\approx 0.04$ m${}^{2}$s${}^{-3}$ and $S_{c\phi }^{a}\approx 0.01$ m${}^{2}$s${}^{-1}$, respectively [9].

Equations (4) and (5) represent the dynamic model of the EKF that governs the prediction step of the SGNSS navigation filter. For the update step, the following measurement vector (for *m* satellites tracked) can be defined as follows:

(6) $$\begin{array}{c} z_{S}={\left[\rho_{a,C}^{1},\rho_{a,C}^{2},...,\rho_{a,C}^{m}\right]}^{T}, \end{array}$$

where the subscript C indicates that the pseudoranges have been partially corrected for common-mode errors, using, for instance, the Klobuchar model [57] to mitigate ionosphere delay, the UNB3 model [10] for tropospheric delay, and satellite clock corrections transmitted in the GPS navigation message [58]. Nevertheless, it is important to acknowledge that the pseudorange measurements mentioned above may still contain residual components of common-mode errors that have not been entirely compensated for by the previously described models.

The SGNSS EKF processes the measurement innovations $\delta z_{S,k}^{-}$ as follows:

(7) $$\begin{array}{c} \delta z_{S,k}^{-}=z_{S,k}-h_{s}\left({\hat{x}}_{S,k}^{-}\right), \end{array}$$

where $h_{s}$ is the nonlinear function that maps the predicted (superscript -) state vector ${\hat{x}}_{S,k}^{-}$ into the estimated (hat symbol) pseudoranges between the rover and satellite, namely

(8) $$\begin{array}{c} h_{s}\left({\hat{x}}_{k}^{-}\right)={\left[{\hat{\rho }}_{a,C}^{1-},{\hat{\rho }}_{a,C}^{2-},...{\hat{\rho }}_{a,C}^{m-}\right]}^{T}, \end{array}$$

with

(9) $$\begin{array}{c} {\hat{\rho }}_{a,C,k}^{s-}=\sqrt{{\left[C_{e}^{I}{\hat{r}}_{es}^{e}-{\hat{r}}_{ea,k}^{e-}\right]}^{T}\left[C_{e}^{I}{\hat{r}}_{es}^{e}-{\hat{r}}_{ea,k}^{e-}\right]}+\delta {\hat{\rho }}_{c,k}^{a-}, \end{array}$$

where *k* is the iteration index, ${\hat{r}}_{es}^{e}$ is the estimated satellite position, ${\hat{r}}_{ea,k}^{e-}$ is the estimated rover position, and $\delta {\hat{\rho }}_{c,k}^{a-}$ is the estimated receiver clock bias.

The measurement matrix is defined as follows:

(10) $$\begin{array}{c} H_{S}^{e}=\left[\begin{array}{cccccccc} -u_{a1,x}^{e} & -u_{a1,y}^{e} & -u_{a1,z}^{e} & 0 & 0 & 0 & 1 & 0\\ -u_{a2,x}^{e} & -u_{a2,y}^{e} & -u_{a2,z}^{e} & 0 & 0 & 0 & 1 & 0\\ \vdots & \vdots & \vdots & \vdots & \vdots & \vdots & \vdots & \vdots \\ -u_{am,x}^{e} & -u_{am,y}^{e} & -u_{am,z}^{e} & 0 & 0 & 0 & 1 & 0 \end{array}\right], \end{array}$$

where $u_{as}^{e}\approx \frac{r_{es}^{e}-r_{ea}^{e}}{∥r_{es}^{e}-r_{ea}^{e}∥}$ and $s\in \left\{\begin{array}{c} 1,...,m \end{array}\right\}$ is the unit line-of-sight vector from rover *a* to satellite *s*.

To weight the uncertainties on the pseudorange observables, the EKF measurement noise covariance matrix can be set to

(11) $$R_{S}=\begin{array}{c} \left[\begin{array}{ccccc} \sigma_{\rho 1}^{2} & 0 & \cdots & 0\\ 0 & \sigma_{\rho 2}^{2} & \cdots & 0\\ \vdots & \vdots & \ddots & \vdots & \\ 0 & 0 & \cdots & \sigma_{\rho m}^{2} \end{array}\right], \end{array}$$

with

(12) $$\begin{array}{c} \sigma_{\rho s}^{2}=\frac{\sigma_{\rho Z}^{2}}{\sin^{2}\left(\theta_{nu}^{as}\right)}, \end{array}$$

where $\theta_{nu}^{as}$ is the satellite *s*’s elevation angle and $\sigma_{\rho Z}^{2}$ is a constant empirical value for the pseudorange uncertainty when the latter is at zenith, which for a 1 Hz EKF update interval is typically around (1–5) m${}^{2}$ [9].

Utilizing the aforementioned system and measurement models, the SGNSS EKF can be implemented using the conventional discrete equations found in the standard literature. For additional details, please refer to [9,12,13,59].

### Relative GNSS Position Estimation

As mentioned in Section 1, to compute the position using the RGNSS approach, the rover needs to access raw observables provided in a timely manner from an available nearby reference station. Similar to Equation (1), the pseudorange taken at the reference station *r* can be modeled as

(13) $$\begin{array}{c} \rho_{r}^{s}=r_{rs}+\delta \rho_{c}^{r}-\delta \rho_{c}^{s}+\delta \rho_{I,r}^{s}+\delta \rho_{T,r}^{s}+\delta \rho_{E}^{s}+\delta \rho_{M,r}^{s}+w_{\rho ,r}^{s}, \end{array}$$

where $r_{rs}$ is the true range from the ECEF position of satellite *s* ($r_{es}^{e}$) to the ECEF position of the reference station ($r_{re}^{e}$).

In the generic case where at least m≥4 satellites are in view of the reference station and rover antennae and are tracked by single-frequency GNSS receivers to which each is connected, the same number *m* of pseudoranges is expected to be tracked. Single-differenced (SD) pseudoranges may be obtained by subtracting the observables of the reference station *r* from those received by the rover *a* in the following equation:

(14) $$\begin{array}{c} \Delta \rho_{ra}^{s}=\rho_{a}^{s}-\rho_{r}^{s}, \end{array}$$

Integrating Equations (1) and (13) into Equation (14) yields the following SD pseudorange model for *m* satellites in view [9]:

(15) $$\begin{array}{c} \left\{\begin{array}{c} \Delta \rho_{ra}^{1}=\rho_{a}^{1}-\rho_{r}^{1}={\left[-u_{a1}^{e}\right]}^{T}r_{ra}^{e}+\Delta \rho_{c}^{ra}+\Delta \rho_{M,ra}^{1}+\Delta w_{\rho ,ra}^{1}\\ \vdots \\ \Delta \rho_{ra}^{m}=\rho_{a}^{m}-\rho_{r}^{m}={\left[-u_{am}^{e}\right]}^{T}r_{ra}^{e}+\Delta \rho_{c}^{ra}+\Delta \rho_{M,ra}^{m}+\Delta w_{\rho ,ra}^{m} \end{array}\right. \end{array}$$

where $u_{as}^{e}$, $s\in \left\{\begin{array}{c} 1,...,m \end{array}\right\}$ is the unit line-of-sight vector from satellite *s* to the antennae and $\Delta \rho_{c}^{ra}=\delta \rho_{c}^{a}-\delta \rho_{c}^{r}$, $\Delta \rho_{M,ra}^{s}=\delta \rho_{M,a}^{s}-\delta \rho_{M,r}^{s}$, and $\Delta w_{\rho ,ra}^{s}=w_{\rho ,a}^{s}-w_{\rho ,r}^{s}$ are the SD receiver clock delay, and multipath and white Gaussian-like measurement noise for satellite *s*, respectively. The GNSS antennae *r* and *a* are considered to be close enough that their line-of-sight vectors to each satellite are parallel.

It should be noticed in Equation (15) that the SD pseudorange measurements are linearly related to the ECEF-resolved baseline vector ($r_{ra}^{e}$ = $r_{ea}^{e}-r_{er}^{e}$) and that they are no longer corrupted by the common-mode errors (($\delta \rho_{c}^{s},\delta \rho_{I,i}^{s},\delta \rho_{T,i}^{s},\delta \rho_{E}^{s}$), $i\in \left\{\begin{array}{c} a,r \end{array}\right\}$), which were expected to be canceled out during the differentiation process. However, this cancellation is valid solely when the the rover is able to access the reference station measurements is in a delimited time span (of minutes, typically) and when the physical separation (baseline) between both antennae is within a certain range. The investigation of both effects on moving agricultural rovers equipped with low-cost GNSS receivers is the main focus of this work.

The following state vector (comprising *n* = 8 states) can be defined for the EKF-based SD RGNSS positioning estimation algorithm:

(16) $$\begin{array}{cc} x_{R}^{e} & ={\left[{\left(r_{ra}^{e}\right)}^{T},{\left(v_{ra}^{e}\right)}^{T},\Delta \rho_{c}^{ra},\Delta {\dot{\rho }}_{c}^{ra}\right]}^{T}\in R^{n}, \end{array}$$

where the subscript *R* denotes the RGNSS, $v_{ra}^{e}$ is the rover velocity relative to the reference station, and $\delta {\dot{\rho }}_{c}^{ra}$ is the rover clock drift, which is also relative to the reference station.

The dynamic model that describes how the states in Equation (16) are propagated forward in time and the associated process noise PSDs are the same as in Equations (4) and (5). Concerning the EKF update step, in turn, the measurement vector (for *m* satellites tracked) comprises the SD pseudoranges; in other words, we have

(17) $$\begin{array}{c} z_{R}={\left[\Delta \rho_{ra}^{1},\Delta \rho_{ra}^{2},...,\Delta \rho_{ra}^{m}\right]}^{T}, \end{array}$$

Similar to Equation (7), the measurement innovations vector that is effectively processed by the EKF is

(18) $$\begin{array}{c} \delta z_{R,k}^{-}=z_{R,k}-h_{r}\left({\hat{x}}_{R,k}^{-}\right), \end{array}$$

with

(19) $$\begin{array}{c} h_{r}\left({\hat{x}}_{R,k}^{-}\right)={\left[\Delta {\hat{\rho }}_{ra}^{1-},\Delta {\hat{\rho }}_{ra}^{2-},...,\Delta {\hat{\rho }}_{ra}^{m-}\right]}^{T}, \end{array}$$

where the SD pseudorange estimates can be computed as follows:

(20) $$\begin{array}{c} \Delta {\hat{\rho }}_{ra}^{s-}=∥C_{e}^{I}{\hat{r}}_{es}^{e-}-{\hat{r}}_{ea}^{e-}∥-∥C_{e}^{I}{\hat{r}}_{es}^{e-}-{\hat{r}}_{er}^{e-}∥+\Delta {\hat{\rho }}_{c}^{ra-}. \end{array}$$

The SD measurement matrix $H_{R}^{e}$, which is the geometric matrix that characterizes the user-satellite geometry, is defined as in Equation (10). Lastly, the measurement noise covariance matrix, which is used to weight the uncertainties of the SD pseudorange observables, is

(21) $$\begin{array}{c} R_{R}=2R_{S}, \end{array}$$

where the factor 2 arises from the SD process.

## 3. Material and Methods

**Figure 1:**
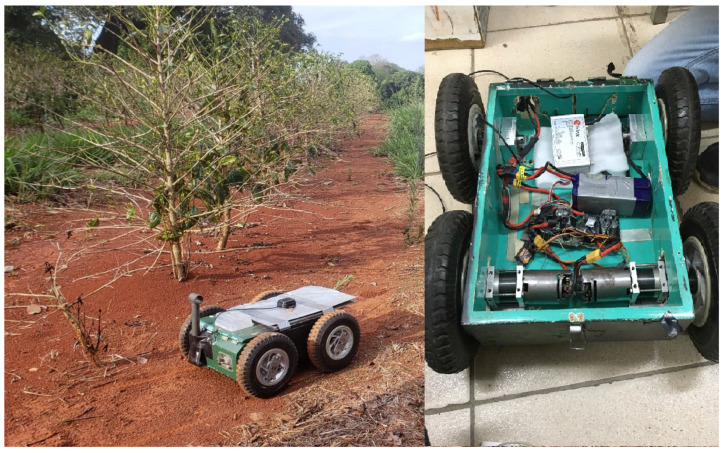
Agricultural rover equipped with u-blox C102-F9R application board and ANN-MB-01 antenna (black box in the gray area over the robot’s roof) used in the experimental test.

In order to analyze the influence of communication failure and baseline separation (between the rover and reference station) on the RGNSS position accuracy, an experimental test was performed. The test consisted of equipping a radio-controlled agricultural rover with a u-blox C102-F9R application board connected to a u-blox antenna (model ANN-MB-01), which was attached to the roof of the rover as illustrated in Figure 1. Despite being a multi-band, multi-constellation receiver, in the test, C102-F9R only acquired single-frequency (L1) coarse acquisition (C/A) pseudorange measurements from the GPS constellation, as these are the sole signals that the majority of mass market low-cost GNSS equipment is currently able to track. Embedded into the rover were also a notebook for collecting the C102 module data via u-center software v22.07, a pair of lithium polymer (LiPo) batteries, electric motors, and electronic speed controllers (ESC) to propel the rover, and a radio control receiver to remotely guide the latter.

### 3.1. Experimental Data Acquisition

**Figure 2:**
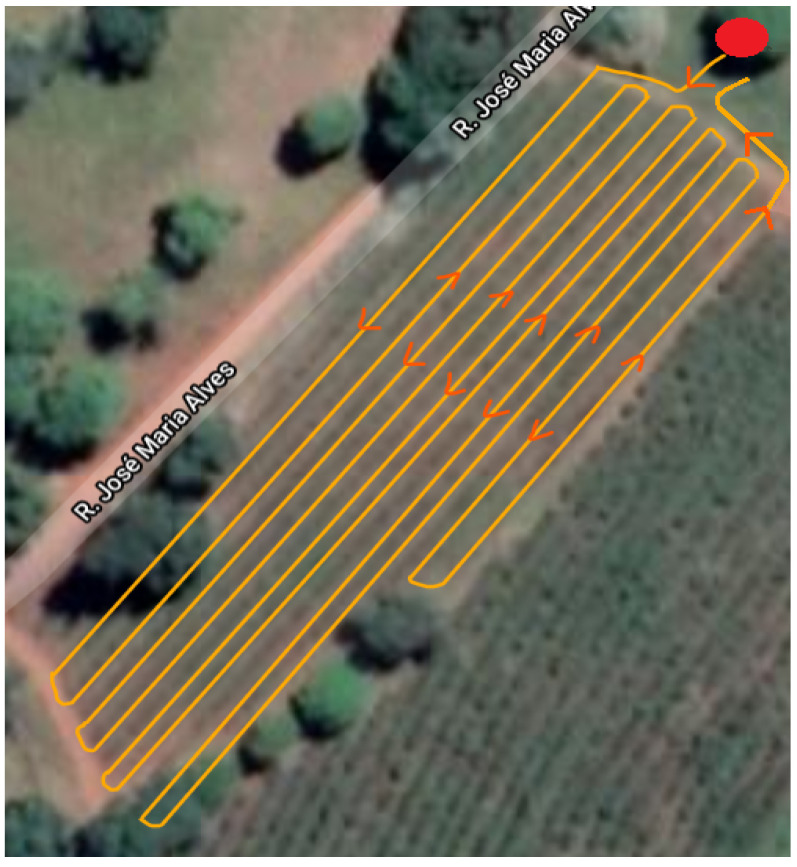
Planned and executed path for data acquisition at coffee plantation located at Federal University of Lavras.

The test was conducted in an experimental coffee plantation composed of young coffee trees with a height of about 1.5 m located at the Federal University of Lavras (UFLA). The rover (Figure 1) was guided through the corridors of the coffee plantation, which are approximately 130 m long, (The planned and executed path is illustrated in Figure 2) collecting and saving, in a personal computer, GPS raw data at a tracking interval of 1 s so that the data could be post-processed (aiming at the rover’s position computation) using the following two algorithms developed in the Matlab^®^ environment:

1. An EKF-based standalone GNSS approach using pseudorange measurements. This approach will be referred to as SGNSS in the ensuing text for the sake of brevity.
2. EKF-based relative GNSS using single-differentiated pseudorange measurements. Conversely, this approach will be referred to as RGNSS hereinafter.

For RGNSS position estimation, the selection of the corresponding reference station was based on the Brazilian Network for Continuous Monitoring of GNSS (RBMC), which is operated and maintained by the Brazilian Institute of Geography and Statistics (IBGE). The RBMC is renowned as the most precise geodetic reference infrastructure in Brazil, comprising 147 geodetic stations strategically distributed across the country, all meticulously positioned with high-precision coordinates. Each station is equipped with a high-performance receiver that continuously collects and stores GNSS observables and navigation data. The RBMC offers GNSS data in three different formats: real-time data are available through the Networked Transport of RTCM via Internet Protocol (NTRIP) “caster” server [60]; post-processed data are provided in Receiver Independent Exchange (RINEX) format (versions 2 and 3) at 15 s tracking intervals; and post-processed data at 1 s tracking intervals are available in RINEX 3-only format [61]. These datasets are freely accessible to all users via the IBGE website [62]. Although some of these bases are equipped with multi-frequency, multi-constellation receivers, only L1 C/A pseudorange measurements from the GPS constellation were used for state estimation during the experimental tests.

### 3.2. Performance Assessment Criteria

In order to determine the reliable ground truth values for the rover position (so that the corresponding estimates could be compared against them), precise real-time kinematics (RTK) positioning using dual-frequency wide-lane carrier phase observables (from both the rover and the closest RBMC station) was determined, whose integer ambiguities were solved and fixed using the least squares ambiguity decorrelation adjustment (LAMBDA) method proposed in [63,64].

We considered three criteria for assessing the position estimation performance: the individual channel position error ($e_{ic_{k}}$), defined in Equation (22); the horizontal position error ($e_{h_{k}}$), defined in Equation (23); and the total position error ($e_{t_{k}}$), defined in Equation (24):

(22) $$\begin{array}{c} e_{ic_{k},N,E,D}=r_{ea,N,E,D}^{n}-{\hat{r}}_{ea_{k},N,E,D}^{n}, \end{array}$$

(23) $$\begin{array}{c} e_{h_{k,l}}=∥\left[\begin{array}{ccc} 1 & 0 & 0\\ 0 & 1 & 0 \end{array}\right]\left(r_{ea}^{n}-{\hat{r}}_{ea_{k}}^{n}\right)∥, \end{array}$$

(24) $$\begin{array}{c} e_{t_{k,l}}=∥(r_{ea}^{n}-{\hat{r}}_{ea_{k}}^{n})∥, \end{array}$$

where $r_{ea}^{n}$ is the NED-resolved ground truth position of the rover and ${\hat{r}}_{ea_{k}}^{n}$ is the corresponding estimate.

## 4. Experimental Results

This section presents the results and discusses the experimental position estimation performance of a low-cost GNSS receiver-equipped moving agricultural rover, considering the degradation effects of communication failure and baseline separation between the latter and the reference station.

### 4.1. Sensitivity to Communication Failure

The first goal was to verify the extent to which the RGNSS estimation algorithm was able to achieve a horizontal accuracy level of 1.5 m at 1σ, as required by the SAE J2945 standard [34]. The EKF algorithm processed the entire batch of GNSS measurements collected during the test ($k=1,...,N_{d}$; $N_{d}=800$ epochs, where 1 epoch corresponds to 1 s) as if they were occurring in real time (i.e., incrementally) to estimate the state vector at each time index *k* for a given value of communication failure *l*, which is also in seconds, using the SD pseudorange $\Delta \rho_{ra}^{s}\left(k;k-l\right)$. The experiment was repeated for values of l=0,...,6000 epochs, which corresponded to a communication failure of up to 100 min. In this extreme situation, the rover used data from a reference station collected 100 min earlier in order to compute its own position at the current epoch.

For comparative purposes, the rover position estimated using the SGNSS approach was also included in the analysis. The reference station chosen for RGNSS position estimation was the MGLA base station, which is part of the RBMC and situated at UFLA’s campus. This particular base station supplied the rover with raw pseudorange measurements. These measurements were utilized to deploy common mode error cancellation during the rover’s own position estimation process. The baseline separation between the rover and the MGLA reference station (i.e., the distance between the base station and the coffee plantation where the test was performed) was estimated to be approximately 1 km. For each fixed value of communication failure *l*, the mean and standard deviation of $e_{ic_{k,l},N,E,D}$, $e_{h_{k,l}}$, and $e_{t_{k,l}}$ were computed from the experimental data by averaging the last $\left(N_{d}-l_{max}\right)$ epochs, where $l_{max}$ is the maximum value for communication failure (6000). Table 1 summarizes the mean and standard deviation of the position error criteria defined in Equations (22)–(24) for communication failure l=0 s (i.e., RGNSS rover position estimation computed using reference station’s pseudorange measurements received at the same epoch as the rover $\Delta \rho_{ra}^{s}\left(k;k\right)$). For comparison purposes, the position error statistics for the SGNSS approach are also included in Table 1.

As one can see in Table 1, in the absence of communication failure, the RGNSS approach presented improved performance in all error criteria if compared with the SGNSS technique since the mean errors of the former were closer to zero, aside from presenting smaller standard deviations. One can also notice that the SGNSS approach was not able to comply with the SAE J2945 standard position accuracy requirements, while RGNSS was. This is an indication that the common mode errors were effectively mitigated.

**Figure 3:**
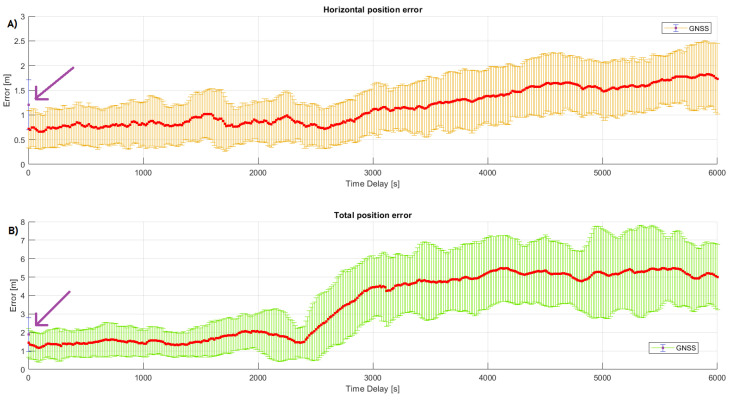
Mean error of horizontal (**A**) and total (**B**) position as function of communication failure. The first plotted data refer to the position estimation performed by the SGNSS algorithm (highlighted by the arrow in purple), and the remaining data (red dots) refer to the RGNSS algorithm.

The analysis that follows investigates the detrimental effect of communication failures between the rover and reference station data, where incremented time delays (in accessing the reference station’s raw data) were purposely added in the position estimation process. Figure 3 shows the mean error along with the plus and minus one standard deviations (indicated by the vertical bars in each point) as a function of the communication failure. The first point (in purple) is the rover position estimation error performed by the SGNSS algorithm, whereas the remaining points (in red) are the position estimation error performed by the RGNSS algorithm.

As can be noticed in Figure 3, the RGNSS performance was better than that of SGNSS, which is in agreement with [49,65,66]. Another outcome from Figure 3 is that the RGNSS horizontal position accuracy remained under 1.5 m at 1σ for communication failures up to 3000 s, which is sufficient to meet the SAE J2945 standard specifications. After this value of time delay, the RGNSS performance became similar to that of SGNSS and became even worse as the communication failure increased, indicating that the common mode error cancellation was no longer valid. For l=6000 s, for instance (i.e., when the rover position estimation was computed using the reference station’s pseudorange measurements received 6000 epochs earlier $\Delta \rho_{ra}^{s}\left(k;k-6000\right)$), the mean horizontal and total position errors were 1.726 m and 4.996 m, becoming 242% and 349% larger than the corresponding errors when there was no communication failure, respectively. The standard deviation also became larger as *l* was incremented. When l=0 s, the standard deviation for the mean position error in the horizontal channel was σ=0.381 m, whereas for l=6000 s, the standard deviation became σ=0.713 m, which was almost two times larger. This shows that the dispersion in the position uncertainties became wider as communication failures increased, which were due to the increasingly incorrect common mode error cancellation (due to the time correlation of the common mode errors).

Table 2, in sequence, summarizes some selected measures of the position accuracy for the horizontal position error, with the communication failure l=0,1500, and 3000 epochs. Column 1 shows the failure in terms of epochs. Column 2 shows the mean error, defined in Equation (23). Column 3 displays the standard deviation. Column 4 reports the maximum value for the horizontal position error. Finally, columns 5 and 6 report the percent of samples that had position errors less than 1 m and 1.5 m, respectively. A recent work by the authors investigated the effects of communication failure on the position estimation accuracy for a static rover [49], and the results showed that a position accuracy of 1 m at 1σ was achievable for failures up to 1500 s. Compared with the results presented herein (Figure 3 and Table 2), one can see that such accuracy at 1σ could not not be achieved, at least not for failures of 1500 s. It is important to highlight, however, the fact that in [49], the position estimation was made using a high-performance GNSS receiver mounted on a static base, while here, a low-cost receiver was employed, mounted on a moving agricultural rover. Those factors have a significant impact on the position accuracy since they affect the quality of the data collected, as suggested in [67].

### 4.2. Sensitivity to Baseline Separation

The second purpose of the experimental test was to analyze the degradation effect of baseline separation on the RGNSS positioning accuracy. As in Section 4.1, the main goal was to identify the maximum baseline separation between the rover and reference station that still allowed the horizontal position accuracy to remain at 1.5 m (1σ).

**Figure 4:**
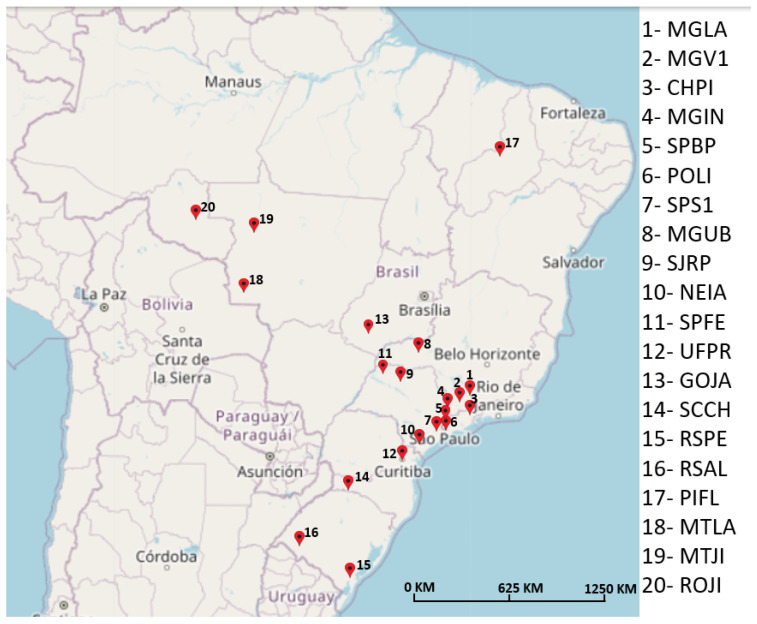
RBMC stations used in the experiment with different baseline distances. The MGLA base station, which corresponds to the location where the test was conducted, is identified by pinpoint 1.

The same GNSS dataset collected in the coffee plantation test was used, and the rover position was again estimated by using the SGNSS and RGNSS approaches. For the latter, 20 RBMC bases were selected to be the reference stations, which provided raw pseudorange measurements for the rover so that the common mode errors could be canceled out during its position estimation process. The reference stations were selected according to their baseline separations from the coffee plantation located at UFLA so that the distances gradually increased in the range from 1 km to 2150 km. Since this was a dynamic test with a moving agricultural rover, the distance between the rover and the reference station was defined using the home point of the experiment (highlighted by the red circle in Figure 2). The chosen bases were MGLA, MGV1, CHPI, MGIN, SPBP, POLI, SPS1, MGUB, SJRP, NEIA, SPFE, UFPR, GOJA, SCCH, RSPE, RSAL, PIFL, MTLA, MTJI, and ROJI, which are displayed in Figure 4. During the experiment, the rover’s trajectory was defined as being toward the MGLA station so that the greatest distance between the latter and the rover for this specific situation was 1 km (rover at the home point).

To analyze the sensitivity of the position estimation accuracy with respect to the baseline separation, the rover position was estimated using the SGNSS and RGNSS algorithms. Each algorithm processed the same set of the rover’s GNSS measurements ($k=1,...,N_{d};N_{d}=800$) as if they were occurring in real time (i.e., incrementally) to estimate the state vector at each time index *k*. For the RGNSS approach, the rover’s position was estimated by considering the selected reference stations. The error criteria defined in Equations (22)–(24) were used to asses the performance of both algorithms.

**Figure 5:**
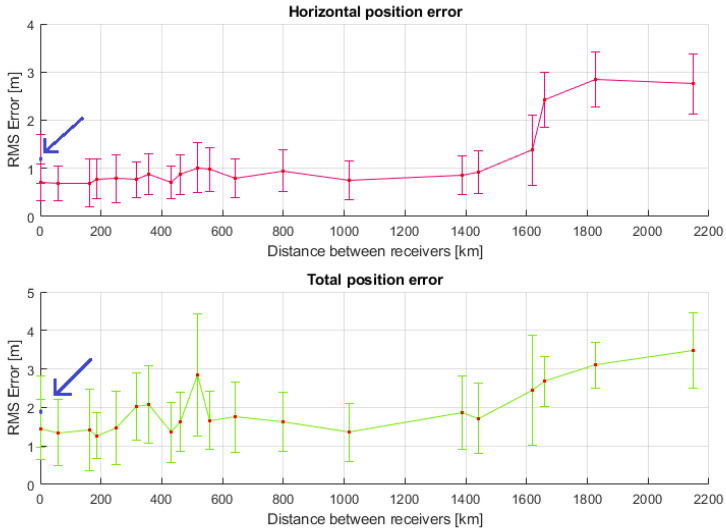
Horizontal position error as function of baseline separation for the RGNSS approach. For a distance of 0 km, the plotted data refer to the position estimation performed by the SGNSS algorithm (highlighted by the arrow in blue).

Figure 5 depicts the mean horizontal and total position error (red squares) as a function of the baseline distances between the receivers. It also displays the standard deviation (vertical bars) of the mean estimated position errors. The first point in the graph (in blue) is the error obtained by using the SGNSS algorithm, whereas the remaining points are the mean errors obtained by using the RGNSS algorithm with different reference stations. As expected, Figure 5 indicates an improvement in the horizontal and total position estimation of RGNSS over SGNSS. The former presented a horizontal position accuracy slightly over 1 m at 1σ when using the first two reference stations (baseline separation of 1.184 km and 60.038 km, respectively), whereas the latter presented a position accuracy of about 1.7 m.

Another interesting outcome from Figure 5 is that the RGNSS approach presented improved performance over SGNSS for the reference stations with a baseline distance of up to 1441 km. For the remaining reference stations (located further away), the position error increased, making the RGNSS performance worse than that of SGNSS. Moreover, the very same reference station with a baseline distance of 1441 km proved to be the limit to which the RGNSS technique was able to comply with the J2945 standard specifications for horizontal position accuracy.

Finally, Table 3 shows the individual distances between the rover and the different reference stations and also summarizes the mean and standard deviations of the horizontal error criteria. A quantitative analysis shows that the mean error increased by 50% (from 0.921 m to 1.377 m) when the reference station was changed from base RSAL to PIFL, located 1441.826 km and 1619.417 km away from the coffee plantation, respectively. This indicates that from this point on, common mode error cancellation was no longer valid, which significantly impacted the position estimation accuracy (a proof of the common mode error spatial correlation).

## 5. Conclusions

This paper addressed the effect of two common degradation factors of the position accuracy of a relative global navigation satellite system (RGNSS): the baseline separation and communication failure between the rover and reference station receivers. A radio-controlled agricultural robot was equipped with a low-cost GNSS receiver to evaluate the performance of the RGNSS technique in a dynamic scenario. A comprehensive review of the main common mode errors that corrupt the GNSS signals was given, as well as an in-depth analytical description of how the RGNSS approach mitigates them.

As with most important verifications from the comprehensive analyses of the effect of communication failures and baseline separation between the rover and reference station, the accuracy of the position estimation with RGNSS was shown to improve in comparison to with the standalone GNSS (SGNSS), especially in the horizontal channel. The timely and spatial correlation of the GNSS common mode errors was demonstrated, and the maximum communication failure and baseline separation required to comply with the SAE J2945 standard specifications were found to be up to 3000 s and approximately 1500 km, respectively, for a moving agricultural rover equipped with a low-cost GNSS receiver.

As suggestions for future works, the authors plan to investigate real-time precise point positioning (RT-PPP) in place of RGNSS, which consists of using precise corrections of common mode errors produced by specialized institutions, such as the International GNSS Service (IGS). Another topic of interest is the integration between inertial navigation systems (INSs) and GNSSs, as they are complementary in terms of advantages and drawbacks. Finally, another type of analysis that is worthy of investigation involves comparing the extended (EKF) and unscented Kalman filter (UKF) approaches in light of the following well-known trade-off: position estimation accuracy versus computation effort.

## Figures and Tables

**Table 1:** Position error statistics for SGNSS and RGNSS approaches.

	SGNSS		RGNSS	
	**Mean** **(m)**	**Std. Dev.** **(m)**	**Mean** **(m)**	**Std. Dev.** **(m)**
North	1.0501	0.498	0.528	0.351
East	0.459	0.371	0.395	0.308
Down	1.293	1.014	1.121	0.866
Horizontal	1.202	0.505	0.713	0.381
Total	1.883	0.925	1.432	0.779

**Table 2:** Horizontal position performance for RGNSS as a function of communication failure.

Failure	Mean	Std. Dev.	Max.	Probability ($e_{h_{k}}$)	
**(epochs)**	**(m)**	**(m)**	**(m)**	**Err < 1 m**	**Err < 1.5 m**
				**(%)**	**(%)**
0	0.713	0.381	2.039	77.32	96.43
1500	0.952	0.471	2.295	56.18	86.24
3000	1.115	0.494	2.961	42.80	79.36

**Table 3:** Horizontal position error statistics for each reference station used by the RGNSS approach. The first row is the position estimation metrics obtained with the SGNSS algorithm.

	Baseline Separation (km)	Mean Error (m)	Std. Dev. (m)
**SGNSS**	Does not apply	1.202	0.505
**MGLA **	1.184	0.713	0.381
**MGV1**	60.038	0.692	0.357
**CHPI**	162.245	0.696	0.503
**MGIN**	185.908	0.782	0.416
**SPBP**	248.682	0.789	0.501
**POLI**	316.228	0.763	0.362
**SPS1**	355.443	0.881	0.426
**MGUB**	429.269	0.703	0.341
**SJRP**	459.137	0.875	0.412
**NEIA**	519.199	1.012	0.517
**SPFE**	558.059	0.976	0.457
**UFPR**	640.374	0.790	0.400
**GOJA**	800.357	0.950	0.442
**SCCH**	1015.505	0.754	0.406
**RSPE**	1389.495	0.851	0.405
**RSAL**	1441.826	0.921	0.448
**PIFL**	1619.417	1.377	0.729
**MTLA**	1658.773	2.430	0.572
**MTJI**	1825.606	2.849	0.576
**ROJI**	2148.614	2.752	0.627

## Data Availability

The dataset generated and analyzed during the current study is available in the FIGSHARE repository at https://doi.org/10.6084/m9.figshare.19946063.v1 (accessed 31 May 2022).
